# Direct observation of quantum percolation dynamics

**DOI:** 10.1515/nanoph-2022-0324

**Published:** 2022-10-21

**Authors:** Zhen Feng, Bing-Hong Wu, Hao Tang, Lu-Feng Qiao, Xiao-Wei Wang, Xiao-Yun Xu, Zhi-Qiang Jiao, Jun Gao, Xian-Min Jin

**Affiliations:** College of Information and Engineering, Wenzhou Medical University, Wenzhou 325000, China; Center for Integrated Quantum Information Technologies (IQIT), School of Physics and Astronomy and State Key Laboratory of Advanced Optical Communication Systems and Networks, Shanghai Jiao Tong University, Shanghai 200240, China; CAS Center for Excellence and Synergetic Innovation Center in Quantum Information and Quantum Physics, University of Science and Technology of China, Hefei, Anhui 230026, China; TuringQ Co., Ltd., Shanghai 200240, China

**Keywords:** femtosecond laser direct writing, localization, quantum percolation, quantum transport

## Abstract

Percolation, describing critical behaviors of phase transition in a geometrical context, prompts wide investigations in natural and social networks as a fundamental model. The introduction of quantum coherence and superposition brings percolation into quantum regime with more fascinating phenomena and unique features, which, however, has not been experimentally explored yet. Here we successfully map these large-scale porous structures into a photonic chip using femtosecond laser direct writing techniques and present an experimental demonstration of quantum transport in hexagonal percolation lattices, probed by coherent light. A quantum percolation threshold of 80% is observed in the prototyped laser-written lattices with up to 1,600 waveguides, which is significantly larger than the classical counterpart of 63%. We also investigate the spatial confinement by localization parameters and exhibit the transition from ballistic to diffusive propagation with the decrease of the occupation probability. Direct observation of quantum percolation may deepen the understanding of the relation among materials, quantum transport, geometric quenching, disorder and localization, and inspire applications for quantum technologies.

## 1 Introduction

Percolation describes an abrupt transition from a disconnected state to connectivity, unveiling the simplest and most fundamental phenomenon in phase transition in statistical physics [1]. The liquid flow in porous media was first studied as a percolation process in 1957 and a most important concept of percolation threshold was proposed to define the critical void fraction where permeation first occurs [2]. Percolation theory reveals a simple rule that structure lattices, with vacant (unavailable) or occupied (available) sites, follow a binomial probability distribution. The seemingly unrelated geometric structure determines a long-ranged correlation in the system at the vicinity of threshold, where rich phenomena occur [1]. This facilitates broad and deep understandings in wide ranges of areas: spanning from natural science (e.g. the fractal coastlines [3], core formation mechanism [4], turbulence [5]) to applied science (e.g. conducting materials [6, 7], colloids [8], magnetic models [9]), and even to social science (e.g. epidemic spreading [10]).

**Figure 1:**
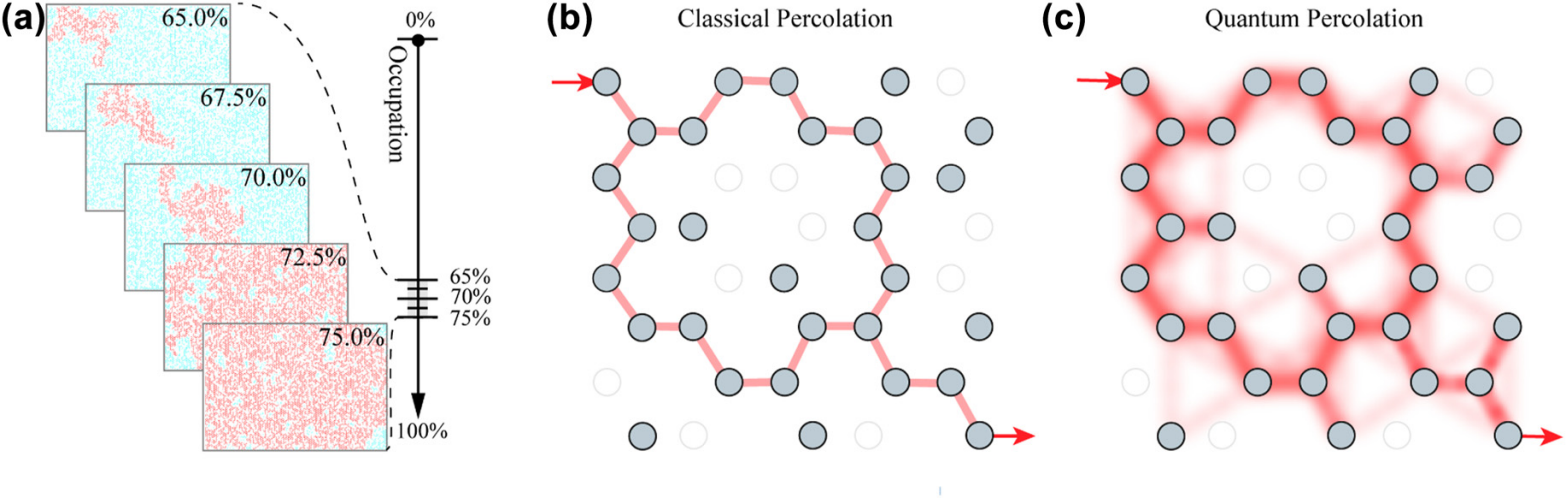
Classical and quantum percolation. (a) Classical percolation is simulated in a hexagonal lattice and the largest cluster is denoted in red. (b–c) A classical particle (no coherence) is injected into the percolation lattice where gray sites are occupied and white ones are empty. A percolation completes when the particle penetrates the lattice and arrives at the other end. In classical percolation (b), an infinite path spanning across the graph means a complete percolation, while in quantum percolation (c), photon can interfere with itself through all possible paths, and can access classically forbidden paths by tunneling.

Transport medium in percolation theory is modeled by regular lattices and a square one is commonly used owing to its simplicity. A hexagonal graph is also a suitable choice when experimental tight-binding approximation and the geometric properties of uniform connectivity are considered [11, 12]. Studying the gradual accretion of nodes or edges in the lattice determine whether the type is site or bond percolation. The former is more general, since every bond model is equivalent to a site one on a different graph, but not vice versa. We therefore are able to investigate general quantum site percolation properties in hexagonal lattices. Each site is occupied independently and randomly with a probability *P* and empty with 1 − *P*. A cluster is identified as a group of connected occupied nearest-neighbor sites, and the largest one is denoted in red in Figure 1(a), where the cluster leads to a large change in connectivity when the occupation probability reaches 70% in a simulated 256, 000-sited hexagonal lattice.

The schematic of a small-scale site percolation in a hexagonal lattice is shown in Figure1(b). A classical picture of penetration means that a cluster spans across from the top left to the bottom right [13]. As its counterpart, quantum percolation is endowed with inherent quantum interference of single or multi particles. Besides, quantum tunneling also exists, which allows particles penetrate through a potential barrier to access a forbidden area. Figure1(c) illustrates a sophisticated process that a quantum particle injected from the top left interferes with all possible paths. The percolation Hamiltonian can be represented by

(1) $$H=t_{1}\underset{<i,j>}{\sum }a_{i}^{†}a_{j}+t_{2}\underset{\left[i,j\right]}{\sum }a_{i}^{†}a_{j}+t_{3}\underset{\left\{i,j\right\}}{\sum }a_{i}^{†}a_{j}+c.c.$$

where <*i*, *j*>, [*i*, *j*], and {*i*, *j*} indicate three types of relations between the occupied site *i* and the nearest-neighbor or the next-nearest-neighbor or the next-to-next-nearest occupied site *j*, and their coupling coefficients are represented by *t*_1_, *t*_2_, and *t*_3_, respectively [14].

Theoretically, quantum percolation Hamiltonian is usually formulated by the first term in the tight-binding approximation. The latter two classically forbidden types of relations, regarded as quantum mechanical tunneling, account for a relatively small fraction. They should be suppressed in the experiment. A hexagonal lattice has the inherent advantage of low tunneling strength relative to other lattice geometries in two dimensions.

In regular lattices without empty sites, a quantum walker can achieve a remarkable speedup over its classical counterpart in virtue of the quantum interference and superposition, such as in one dimensional ordered chain [15], square lattice [16], and glued tree [17]. Disorder, however, can induce a transition from quantum ballistic to classical diffusive transport in a quantum walk, and therefore suppress the expansion of the quantum mechanical wave packets exponentially [18, 19].

Anderson model randomly imposes a small amount of disorder *ϵ* on all sites, and the interference between multiple-scattering paths results in localization. The Hamiltonian is given by $H=\underset{i}{\sum }\epsilon_{i}a_{i}^{†}a_{i}+\underset{i}{\sum }\underset{j}{\sum }t_{ij}a_{i}^{†}a_{j}$ [20]. Quantum percolation with quenched geometric disorder is essentially different from Anderson localization. Empty sites act as high-potential barriers and form many boundaries in the graph. Superposition of quantum particles in different paths also leads to localization, which suppresses the wavepacket spreading.

**Figure 2:**
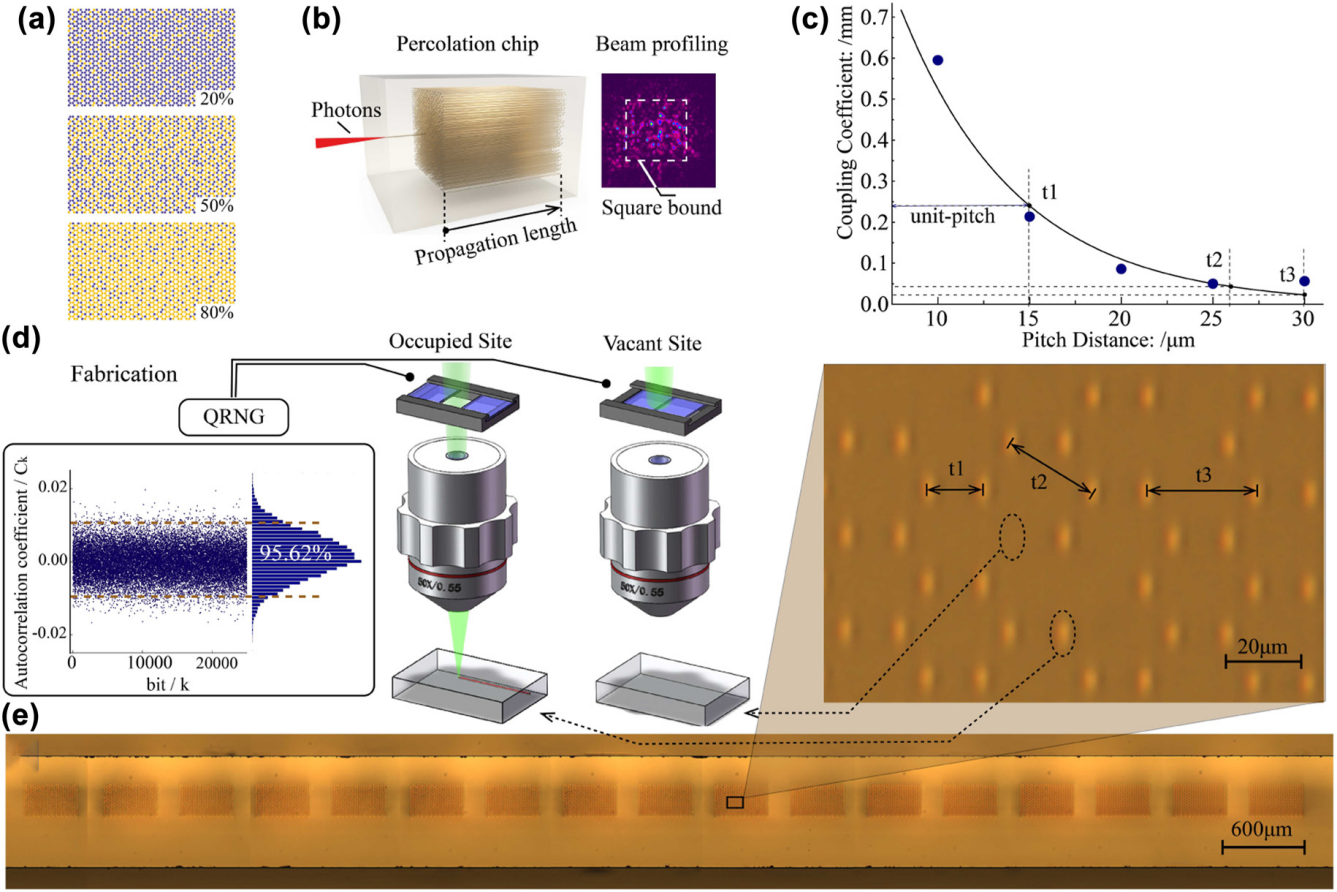
Constructing large-scale percolation lattices on a photonic chip (a) schematic lattices with different occupation probabilities. Yellow and purple sites denote occupied and vacant respectively. (b) Schematic of the quantum percolation experiment. Photon injected into a central waveguide evolves in the percolation waveguide structure. The transverse distribution at the output is collected by an optical beam profiler. (c) The relation between pitch distances and coupling coefficients. The blue dots are experimental data. (d) Programmable fabrication of percolation lattice. A shutter is linked to a quantum random number generator (QRNG) to control the writing laser on and off. The independence and randomness of occupied distribution are ensured when the random number can well pass the autocorrelation test. (e) The cross-sections of seventeen 85%-occupied percolation lattices are pictured. The inset shows the zoomed picture for part of a lattice.

Quantum coherence and superposition associated with graph geometries endows quantum percolation [21] important roles in understanding quantum transport in condensed matter and material physics, including fascinating phenomena such as giant Hall effect [22], quantum phase transitions [23], colossal magnetoresistance in perovskite manganites [24], Anderson localization [18], fractals [25], and photosynthesis [26, 27]. Quantum percolation model has been comprehensively investigated in theory [21, 28], [29], [30], [31]. However, it is still experimentally challenging to realize a large-scale quantum network and the required random quenches with a controllable fashion (Figure 2(a)). In addition, a proper analogue and comparison to classical percolation remain to be built.

## 2 Results

### 2.1 Percolation structure fabrication in a photonic chip

We manage to demonstrate an experimental quantum percolation and directly observe quantum transport transition in hexagonal percolation lattices by successfully mapping these large-scale porous structures into a photonic chip. The lattices with different occupation probabilities (Figure 2(a)), containing 1, 600 static sites for each, are successfully mapped into a photonic chip in a controllable and programmable fashion. Schematic of experimental setup is shown in Figure 2(b). For a quantum walk evolving along the waveguide, the longitudinal propagation length *z* is proportional to the evolution time *t* (*z* = *ct*, where *c* is the light speed within the chip substrate), and hence we use the propagation length *z* to measure the time evolution instead.

The evolution of photons is governed by the Hamiltonian H and the wavefunction at time *z* satisfies:

(2) $$\left|\Psi \left(z\right)\right\rangle =e^{-iHz}\left|\Psi \left(0\right)\right\rangle$$

where $i=\sqrt{-1}$ and $\left|\Psi \left(z\right)\right\rangle$ indicate the probability distribution of finding the quantum walker at each waveguide in the propagation length *z* [32]. In this way, the time evolution in a two-dimensional graph can be mapped into a three-dimensional photonic lattice with an invariant graph along the propagation axis. We realize the three-dimensional prototyping and sophisticated parameter control by using femtosecond laser direct writing [33–36]. Fabrication details are in Supplementary Section 1.

Since all the occupied waveguides are prototyped in a constant velocity, they have the same propagation constant (on-site energy) [37], which means the diagonal elements of the Hamiltonian matrix are identical, thus can be omitted. To make the actual experiment approach to the theoretical model with Hamiltonian $H=t_{1}\underset{<i,j>}{\sum }a_{i}^{†}a_{j}+c.c$ ., we make two experimental efforts on reducing the tunneling effect. One is to explore the evanescent field coupling between the next-nearest-neighbor waveguides [14]. The coupling coefficients in the lattice structure are measured via the coupled mode approach [38] and plotted in Figure 2(c). We can also estimate the ratio of the nearest-neighbor and the next-nearest-neighbor coupling coefficients of hexagonal lattice (0.179) and square lattice (0.396). The other experimental effort is to utilize the lattice with hexagonal geometry, which possesses the inherent advantage of low tunneling strength between next-nearest neighbors in two dimensions.

Percolation lattices are composed of randomly-occupied and -empty sites. As shown in Figure 2(d), a quantum random number generator is embedded to control the laser whether to process the waveguide or to leave it vacant. By this method, we fabricate a series of 40 × 40-waveguide percolation structures (Figure 2(e)).

The probability distribution of a quantum walker at the single-photon level can be successfully reproduced by the intensity distribution of coherent light in the photonic lattice [39]. In this way, we utilize the continuous-wave 810-nm semiconductor laser with spectral linewidth <0.5 nm as the light source to implement a quantum walk, which mainly depends on the first-order interference of light evolving in a lattice [39]. The generated photons are then injected into the central waveguide of percolation lattice using 20× objective lens. After evolving over a full propagation length of 20 mm in the lattice, the outgoing photons are collimated with a 10× microscope objective. The formed transverse spatial pattern is captured by the optical beam profiler for further experimental analysis.

To characterize the experimental results, we extract the light intensity of each photonic waveguide in the pattern with Imread function in Matlab. We build a mask of the all-present hexagonal lattice (pitch: 15 μm; site diameter: 5 μm, adjustable), where each site is constructed by the rectangle function in Matlab with the ’Curvature’ [1, 1] and four position-related parameters. After that, we map the mask onto the patterns to extract the light intensity. If the pattern is an experimental result, we have to consider the lens aberrations and spatial mismatches of each pattern, such as the scaling, rotation and shift. We also introduce some corresponding parameters of central position (*o*_*x*_, *o*_*y*_), horizontal or vertical scaling (*t*_*x*_, *t*_*y*_) and the rotation *θ* to have a perfect overlap between the experimental pattern and the mask. We then extract its light intensities of all 1, 600 sites and normalize them. To eliminate the influence of background noises, the light intensity below 0.02 in waveguide is ignored.

### 2.2 Time evolution characterization of quantum and classical percolation

In the theoretical simulations of quantum percolation, we first build a hexagonal site percolation lattice and formulate the Hamiltonian with the same experimental coupling coefficients. The probability distribution at different propagation length is calculated from Eq. (2). In order to provide a classical counterpart for a fair comparison, we design a theoretical model of classical percolation in the same lattice structure and the detailed mathematic derivation and data analysis are provided in Supplementary Section 2.

To explore the confinement property in each occupation probability, we introduce inverse participation ratio (IPR), which takes almost full information about the Hilbert space and is described mathematically by

(3) $$IPR=\frac{\underset{i}{\sum }|\psi_{i}{|}^{4}}{{\left(\underset{i}{\sum }|\psi_{i}{|}^{2}\right)}^{2}}$$

where |*ψ*_*i*_|^2^ represents the intensity distribution of the transverse section [18]. A state localized in one site is the maximum *IPR* = 1, and it spreading over *N* sites approximately has the minimum *IPR* = 1/*N*. 1/*IPR* is also commonly used and recognized as the IPR by the academic community [21, 40]. In this work, *IPR* has the units of inverse area, thus an average effective width *ω*_eff_ = ⟨*IPR*⟩^−1/2^ represents the ensemble-averaged effective width measured at the lattice output over multiple experiments of the same occupation probability [18, 41].

**Figure 3:**
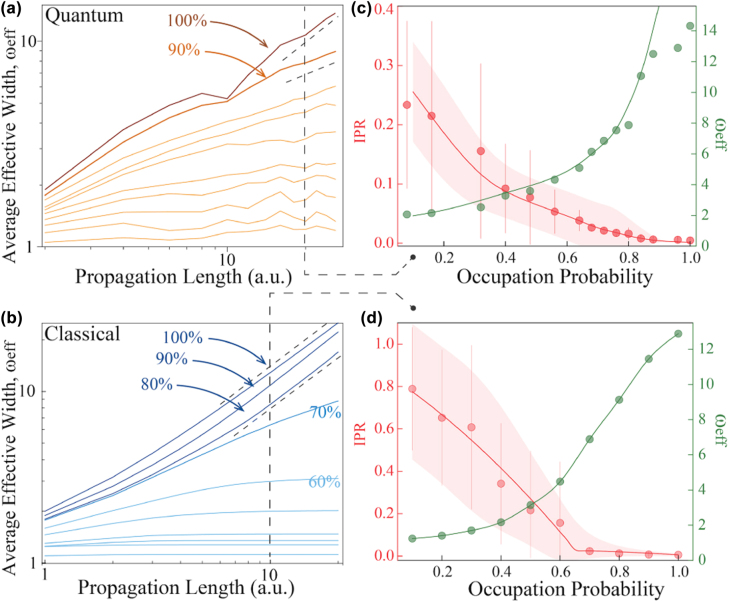
Evolution features in quantum and classical percolation. (a–b) Time evolution of average effective width. (a) Different curves represent different *P* values and the slope $\nu =1,\nu =\frac{1}{2}$ show a ballistic and a diffusive transport respectively. It is localized when $0<\nu <\frac{1}{2}$ . (b) The slope remains at 1 when *P* > 80% and drops rapidly under 70%, showing a great accordance with the predicted threshold at 69.62% and also with 63% in our model. In the propagation length of 20 mm, we retrieve the different distributions of inverse participation ratio (IPR) and effective widths *ω*_eff_ in the (c) quantum and (d) classical percolation. All the points in (c) are experimental data, which are extracted from the transverse spatial patterns captured after photons evolve over 20 mm in percolation lattices. All the points in (d) are from theoretical simulations.

We average *ω*_eff_ from tens of transverse intensity distributions as a function of propagation length to investigate the time evolution of quantum and classical percolation. The results are shown in Figure 3(a) and (b) with each curve representing a different occupation probability from 10 to 100% spacing 10%. It is obvious that average effective widths increase monotonically with propagation length and expand with a higher occupation probability. We set the coordinate axis as a double-logarithmic scale to indicate the power-law relation *ω*_eff_ ∝ *z*^*ν*^, where *ν* is the slope of the curve [18]. In quantum percolation model, photons perform a ballistic transport in an all-present hexagonal lattice and asymptotically approaches the upper dashed line *ν* = 1. With some sites substituted by vacancy, the decrease of the slope *ν* indicates that the broadening is suppressed in different levels: photons have a diffusive spreading with $\nu =\frac{1}{2}$ for *P* = 90% and a gradual evolution of localization with $\nu <\frac{1}{2}$ when occupation probability decreases. On the other hand, the intercepts with the *Y* axis are monotonic to the occupation probabilities, which conform to the mathematical expectation in statistics.

We then fix the propagation length of 20 mm and look into the localization property in each occupation probability. We calculate *IPR* and *ω*_eff_ in the quantum scenario, shown in Figure 3(c). The *ω*_eff_ expands almost exponentially with the increase of the occupation probability. Experimental results denoted by circles match well with the simulated lines. The slight deviation in the high-occupation region is attributed to the fact that photons reach the outer edge due to limited lattice scale. In this figure, *IPR* is a downward convex function, indicating confinement is not very tight under a high-occupation probability *P*. It is distinctly different from the upward convex function in Anderson localization, indicating a system that is sensitive to small disorder [18]. We also observe this downward convex trend in the theoretical study on two-dimensional quantum percolation tight-binding model in square lattice [40]. Figure 3(d) shows the confinement properties of classical percolation in the propagation length of 20 mm from simulated results. We can see that *IPR* approximately fits a quadratic polynomial to the marked points and drops to the base at about 63% and then keeps flat, which reveals a percolation threshold via the turning point in this model. The relatively low initial IPR value in the quantum percolation is further explored and explained in Supplementary Section 3. In addition, the measured standard deviation Δ*IPR* (noted as error bars) is found at the same order of ⟨*IPR*⟩. This result conforms with the prediction that Δ*IPR* is inversely proportional to the occupation probability and the ratio of $\frac{\Delta IPR}{\left\langle IPR\right\rangle }$ is close to unity [42]. Thus, a relatively large error bar with inverse proportion to *P* is expected.

### 2.3 Experimental observation of quantum percolation threshold

To find quantum percolation threshold, we first determined a percolation boundary centered where photons inject, and then establish criteria of penetration that a given proportion of the injection intensity percolates outside the boundary. In this work, we select the square bound with a side length of 16 unit-pitches out of a 40 × 40-waveguide lattice for ease of experimental implementation (a unit-pitch is the spacing between nearest-coupling waveguides; Figure 2 (b) and (c)). The given proportion is set as 10% along the longitudinal propagation length of 20 mm correspondingly. Different combinations of the proportion and the propagation length do not change the nature of percolation and they only result in a small threshold shift along the axis of occupation probability.

**Figure 4:**
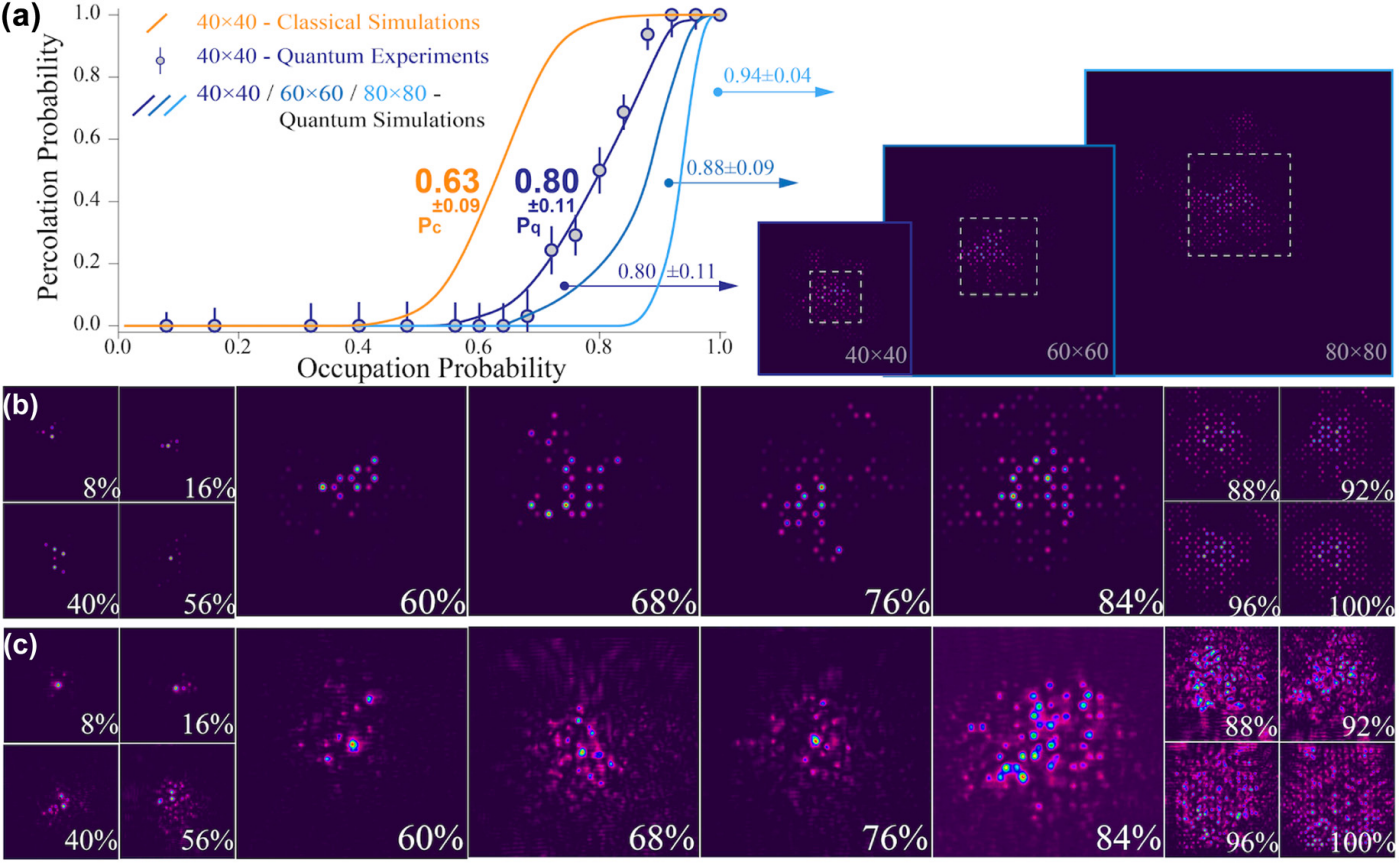
Experimental observation of a transition in quantum percolation. (a) Experimental and theoretical results both demonstrate a sharp quantum percolation transition in the occupation probability range of 80 ± 11%, i.e. from 69 to 91%. Error bars are obtained by error transfer function, see derivation in Supplementary Section 4. With the increase of lattice size, the transition turns sharp and the threshold shifts to the right. The cross sections in each scale are attached. Transverse sections for different occupation probabilities are shown in (b) simulations and (c) experiments.

We gather about 40 individual experimental implementations for each occupation probability and analyze them one by one. We extract the light intensity of each photonic waveguide from the transverse spatial pattern and calculate the proportion outside the boundary. It completes penetration when the proportion is over the given 10%. The success rate of permeation through lattices for each occupation probability is defined as percolation probability. We also generate 100 simulated results for each occupation probability and form a smooth simulation curve. From statistical and visual results retrieved in a 40 × 40 hexagonal lattice (Figure 4(a–c)), we can see a clear quantum percolation transition centered at 80%, which is much higher than the results of 63% obtained from the classical percolation model. The transition is not very sharp, which is attributed to the restricted lattice size.

We first define the span length as the experienced range of occupation probability *P*, where the percolation probability rises from 10 to 90% with a fixed size of hexagonal lattices. In our experiment, we have achieved a size up to 40 × 40, which leads to a span length of ±11%. We then enlarge the lattice size to 60 × 60 and 80 × 80 as well as the corresponding scaling of propagation length and the bound size, see detailed parameters in Table 1). As shown in Figure 4(a), a significant trend has been observed that the thresholds move further to the right and the span lengths become shorter with the size growing. We believe that *P*_*q*_ is up to 1 in our experimental configurations when the size of the lattice tends to be infinite, which is almost intractable to simulate in such model.

**Table 1:** Simulation parameters in different-sized lattices.

Lattice size	Propagation length (mm)	Square bound size
40 × 40	20	16 × 16
60 × 60	30	24 × 24
80 × 80	40	32 × 32

## 3 Conclusions and discussions

In conclusion, we build percolation graphs freely by Hamiltonians engineering and site manipulation, and directly observe quantum transport transition in on-chip large-scale hexagonal lattices, probed by coherent light. The measured quantum percolation threshold of 80% in lattices with up to 1,600 waveguides is significantly larger than the classical counterpart of 63%. We also observe a transition from ballistic to diffusive and then to localized transport with the decrease of occupation probability. Our approach provides a tool for statistical exploration with the established criterion in future experimental research on direct observation of quantum percolation dynamics. It may open the door to explore rich percolation-involved phenomena for quantum simulation, and inspire applications for analog quantum computing. With greater detection capability and photon manipulation in the future, we can probably explore multi-photon quantum percolation. Some future perspectives of the quantum percolation platform are provided in Supplementary Section 5.

Finding exact percolation thresholds has long been attracting enduring explorations for mathematicians because of its fundamental and wide-ranging importance. Early mathematic foundation was established to solve the simplified general Potts model accurately [43], which facilitated many advances in the development of percolation theory, such as scaling relations [44, 45], the hull [46] and renormalization group theory [47]. Percolation threshold is considered as a fundamental characteristic of percolation theory and its value generally depends on the structure parameters (e.g. lattice parameters and dimensionality) [48]. Our work provides an alternative approach to solve the threshold problems by building a percolation simulator, and also a technical means to produce artificial phenomena by intentional acts of the researchers.

## Supplementary Material

Supplementary Material Details
