# Supplementary material for: Direct observation of quantum percolation dynamics
Source: Nanophotonics. 2022 Oct 21;12(3):559–67. doi: 10.1515/nanoph-2022-0324 (PMC11501899; doi:10.1515/nanoph-2022-0324)
Supplement: Supplementary file 1 — Supplementary Material Details [file j_nanoph-2022-0324_suppl.pdf]

Zhen Feng, Bing-Hong Wu, Hao Tang, Lu-Feng Qiao, Xiao-Wei Wang, Xiao-Yun Xu, Zhi-Qiang Jiao, Jun Gao, and Xian-Min Jin*

Supplementary Materials: Direct Observation of Quantum Percolation Dynamics

https://doi.org/10.1515/sample-YYYY-XXXX
Received Month DD, YYYY; revised Month DD, YYYY; accepted Month DD, YYYY

1 Fabrication of Percolation Lattices on a Photonic Chip

A femtosecond laser ($10W, 1026nm$) with $290fs$ pulse duration and $1MHz$ repetition rate is frequency doubled to $513nm$ and directed into an spatial light modulator(SLM) to create burst trains which is focused on a borosilicate substrate ($20mm \times 20mm \times 1mm$) with a $50\times$ objective lens with a numerical aperture of 0.55. A single-mode waveguide is fabricated with a series of optimal laser parameters at a substantially constant temperature and humidity. We prototype a waveguide by 10 nm positioning accuracy with margin mode of SetBack (for acceleration) and Return (for deceleration) to avoid too-high pulse overlap at beginnings and ends of marking trajectories. Whether a waveguide is written or not, is programmably controlled by a quantum random number generator (QRNG), which allows the design of arbitrary porous structure.

About 40×40 straight waveguides are fabricated at a constant velocity of $10mm/s$ for each percolation lattice, i.e. there are 40 layers in the borosilicate substrate with 40 waveguides in each layer. The pitch between two nearest-neighbor waveguides is $15\mu m$. A photonic lattice spans as large as $400\mu m$ in depth and great efforts have been made to process depth-independent single-mode waveguides through power and Spatial Light Modulator (SLM) compensation. In addition, long fabrication duration brings difficulties in highly stable laser output and high environmental stability.

As for the light source to perform experiments, we use the continuous-wave 810-nm semiconductor laser coupled with polarization maintaining mode fiber. The laser specifications include spectral linewidth $<$0.5nm, coherence length $>$419μm, mode field diameter: 4.5um. Photons generated from the 810-nm laser pass a polarized beam splitter and vertically polarized photons are injected into the central waveguide of percolation lattice using $20\times$ objective lens.

2 Classical Percolation Model in Hexagonal Lattices

Here we construct a classical hexagonal percolation model for a fair comparison of our quantum counterpart. A modest amount of viscous liquid is pumped from a central pipe into the hexagonal percolation pipe network where pipes connect every two nearest occupied sites, assuming that it flows at a constant velocity in the network. It shares the same analysis as our quantum percolation model and can help gain insight into the percolation in quantum regime.

We start with the introduction of inverse participation ratio (IPR) to our classical model by

$$IPR = \frac{\int I^2_{(x,y,L)} dxdy}{[\int I_{(x,y,L)} dxdy]^2} = \frac{\sum^N c^2}{(\sum^N c)^2} \tag{1}$$

where c, a constant value, represents occupied sites of viscous liquid and 0 is for vacant site. N denotes the number of occupied sites covered by viscous liquid. Average effective width ω_{eff} can be derived

$$\omega_{eff} = \langle IPR \rangle^{-\frac{1}{2}} = \sqrt{\frac{(\sum^N c)^2}{\sum^N c^2}} = \sqrt{\frac{N^2c^2}{Nc^2}} = \sqrt{N} \tag{2}$$

In this two-dimensional model, it is apparent that the number of sites M is proportional to t^2 in consideration of occupation probability P mentioned in the main text, t is a propagation step. We can obtain the relation:

$$N = PM \propto Pt^2 \tag{3}$$

Then we can get ω_{eff} as follows:

$$\omega_{eff} = \sqrt{N} = k\sqrt{P}t \tag{4}$$

where k is a constant factor. We sketch the relation between propagation step t and average effective width ω_{eff} in a double-logarithmic scale:

$$\log \omega_{eff} = \log k\sqrt{P}t = \log t + \frac{1}{2}\log P + Const. \tag{5}$$

In this system, we derive the relation between propagation step and average effective width (Extended Data Fig. 1). We find liquid expands in a ballistic manner, following our theoretical evolution $\omega_{eff} = \sqrt{PM}z$, as long as $P > 70\%$. When $P \leq 60\%$, a bit lower than the derived classical threshold 63%, the liquid will be trapped in the pipes.

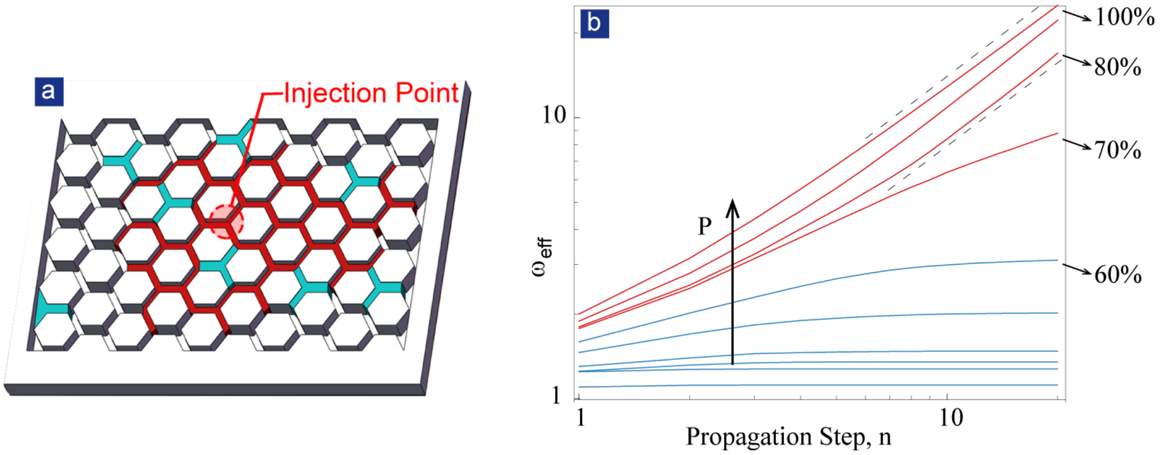

Fig. 1: Classical Percolation Model in Hexagonal Lattices. **(a)** Sketch of hexagonal percolation pipe network. Viscous liquid is pumped into the injection point and evolutes in available paths marked in red. **(b)** Time evolution in classical percolation model. Three regions are separated by the occupation probability (i) $P > 70\%$, the slopes ν is going to approach 1; (ii)$60\% < P < 70\%$, liquid leads to a relatively slow expansion; (iii) $P \leq 60\%$, liquid is trapped in the pipes.

3 Estimation of Maximal Values in IPR

Inverse Participation Ratio (IPR) measures the fractional size of the wave packets spreading across the lattice [1]. It is influenced by disorder [2] and is often used to assess localization in the thermodynamic limit [3]. According to Eq.(2), IPR is of the same order as $1/i$ if the wave packets spread over i sites. A classical particle can only access to the nearest neighbor sites in one direction at a time, while a quantum particle is

allowed to simultaneously tunnel into next-to-nearest neighbors or next-to-next-nearest neighbors. Therefore, quantum particles in the input site have the possibility to jump over 12 candidate sites, while 3 for classical particles (see Fig.2).In Fig.3(c)&(d) in the main text, the leftmost data point is situated at the occupation rate of 8%. In a rough consideration of one-step hopping, quantum particles take $1 + 0.08 \times 12 = 1.96$ sites (IPR ≈ 0.5), since occupation of each sites follows the binomial distribution. Classical particles take $1 + 0.08 \times 3 = 1.24$ sites, which means IPR is about 0.81. Experimental inevitable noise in quantum percolation further moves the IPR down. The qualitative analysis shows the quantum percolation evolution has a relatively low initial IPR value and it conforms with the theoretical researches [1, 3].

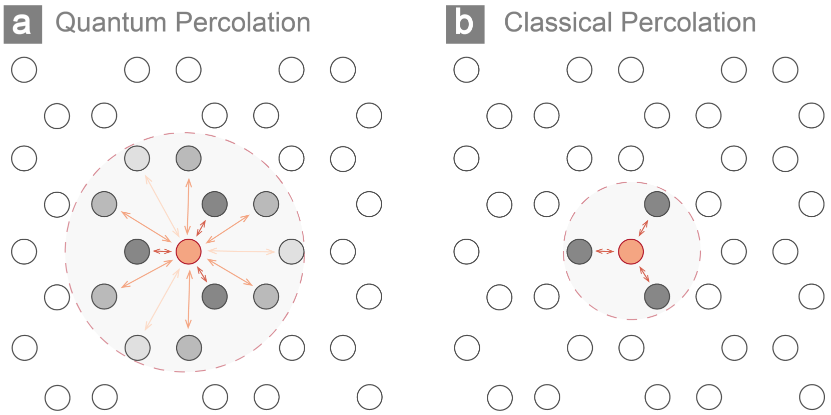

Fig. 2: One-step hopping of **(a)** quantum and **(b)**classical particles from the input site in the hexagonal percolation lattices.

4 Statistics of Quantum Percolation Threshod

In our experiments, we gather N_P individual experimental results for each occupation probability and count the number n of complete percolations from all these N_P processed data of each value P. The fraction of complete percolations Pr is defined by

$$Pr = \frac{n_P}{N_P}(P \text{ is a given occupation probability}) \tag{6}$$

In this case, we expand the expression Pr as:

$$Pr = \frac{\sum_i^{N_P}(P_i)}{N_P} \tag{7}$$

where P_i is 1 when the ith experiment percolation is accomplished, otherwise is 0; $\sum_i^{N_P}(P_i) = n$ ($P_i = 0$ or 1). Independent and identically distributed random variables $P_1, P_2, \cdots, P_i, \cdots$ follows Bernoulli distribution with the occupation probability P:

$$\Delta P_i = \sqrt{P(1-P)} \tag{8}$$

The error bars are derived by the error transfer function:

$$\Delta Pr = \sqrt{\sum_i^{N_P}(\frac{\partial Pr}{\partial P_i})^2(\Delta P_i)^2} = \frac{\Delta P}{\sqrt{N_P}} = \frac{\sqrt{P(1-P)}}{\sqrt{N_P}} \tag{9}$$

5 Quantum Percolation Platform

Porous structures are important in many areas including biological systems, condensed matter and material physics. Understanding the nature and dynamics in porous structure is of great interest. Transport in bulk media is mimicked by the propagation of photons in a photonic lattice and randomly absent sites can constitute pores in percolation structure. We provide an effective and practical experimental platform to build scalable percolation graphs freely by engineering Hamiltonians and manipulating photonic lattices. A site is fabricated in a controlable and programmable fashion to modulate the connectivity and coupling coefficients t_{ij} between two sites i and j.

This platform offers an opportunity to directly observe quantum percolation dynamics, laying the basis for measuring critical exponents in quantum systems. Issues on how the critical scaling exponents may be affected by quantum effects are interesting open questions and deserve further investigations. The method could become a standard experimental approach in future statistical exploration. It is a first step of experimental quantum percolation, and also a crucial step towards high complexity associated with many open questions by involving more particles and dimensions. For instance, Hong-Ou-Mandel interference will become dominant in multi-particle quantum percolation, which cannot be simulated by coherent light, and even cannot be predicted by the most powerful supercomputer, known as quantum supremacy based on boson sampling on percolation lattices.

References

[1] Hul, O., Šeba, P. & Sirko, L. Departure of some parameter-dependent spectral statistics of irregular quantum graphs from random matrix theory predictions. *Phys. Rev. E* **79**, 66204 (2009).

[2] Johri, S. & Bhatt, R. N. Singular behavior of eigenstates in Anderson's model of localization. *Phys. Rev. Lett.* **109**, 76402 (2012).

[3] Thomas, B. S. D. & Nakanishi, H. Two-dimensional quantum percolation with binary nonzero hopping integrals. *Phys. Rev. E* **94**, 42141 (2016).
